# A retroperitoneal urinoma secondary to metastatic urothelial carcinoma mimicking psoas muscle malignancy: Case Report and literature review

**DOI:** 10.3389/fonc.2025.1708321

**Published:** 2026-01-12

**Authors:** Tianping Li, Haijuan Lv

**Affiliations:** Department of Radiology, the Second Affiliated Hospital of Jiaxing University, Jiaxing, China

**Keywords:** CT, metastasis, retroperitoneal, urinoma, urothelial carcinoma

## Abstract

**Purpose:**

This study aims to improve the understanding of imaging features of urinoma associated with urothelial carcinoma metastasis, enhance preoperative diagnostic accuracy, and reduce misdiagnosis rates.

**Methods:**

A 71-year-old male was admitted for evaluation following the incidental imaging detection of a left ureteral mass during an outpatient visit over the preceding 10 days. The patient was asymptomatic. Physical examination revealed a blood pressure of 195/88 mmHg, with no tenderness in either renal region. Laboratory investigations showed normal urinalysis and urinary cytology, with no tumor cells observed in urine smears. Contrast-enhanced CT urography (CTU) was subsequently performed.

**Results:**

Contrast-enhanced CT revealed a large cystic-solid mass in the left retroperitoneal and psoas muscle region, with poorly defined margins relative to the left kidney. The left ureter traversed the lesion and exhibited enhancement within the solid components, raising suspicion for a low-grade malignant tumor originating from the psoas muscle and involving the left kidney and ureter. Surgical pathology confirmed an invasive high-grade urothelial carcinoma of the left ureter, with infiltration into retroperitoneal cysts, the adjacent kidney, and the psoas muscle. Six months postoperatively, the tumor recurred and progressed rapidly, with evidence of widespread metastatic spread.

**Conclusion:**

CTU plays a crucial role in diagnosing urothelial tumors, though its accuracy may be limited in patients with kidney dysfunction. The characteristic course of the ureter traversing a mass, along with hyper enhancing solid components at the obstructed distal ureter and adjacent tissues, can assist in accurate diagnosis and help reduce the risk of misdiagnosis. However, surgery for locally invasive tumors risks spreading cancer cells and worsening outcomes.

## Introduction

A urinoma is defined as an encapsulated collection of urine outside the urinary tract, resulting from disruption of the collecting system and lacking an epithelial lining (1, 2). Common causes of spontaneous ureteral rupture include urinary tract obstruction, iatrogenic injury, and trauma (3). Obstructive uropathy may be caused by urinary calculi, tumors, congenital stenosis, or external compression. However, urinomas secondary to ureteral malignancies are rarely reported. In this case, we present an asymptomatic giant retroperitoneal urinoma with tumor seeding, caused by urothelial carcinoma of the left ureter.

## Case presentation

A 71-year-old male presented with unexplained weight loss and was admitted to the hospital following the incidental detection of a left ureteral abnormality on CT imaging performed over 10 days prior. The patient was asymptomatic, and his physical examination at that time was unremarkable, reporting no discomfort, flank pain, chills, fever, or lower urinary tract symptoms. He denied any history of trauma or prior surgery.

### Laboratory and physical examination

During hospitalization, the patient’s laboratory and physical examination findings were as follows: Tumor markers: squamous cell carcinoma antigen 1.61 ng/mL, ferritin 386.2 ng/mL. Urine analysis: α1-microglobulin 14.47 mg/L. Urine cytology showed no malignant cells. Renal function: Serum creatinine and blood urea nitrogen levels were within normal limits. Electrolyte levels were normal. Vital signs and physical examination: Blood pressure was elevated at 195/88 mmHg, and no tenderness was noted over either renal angle.

### CT examination

**Figure 1:**
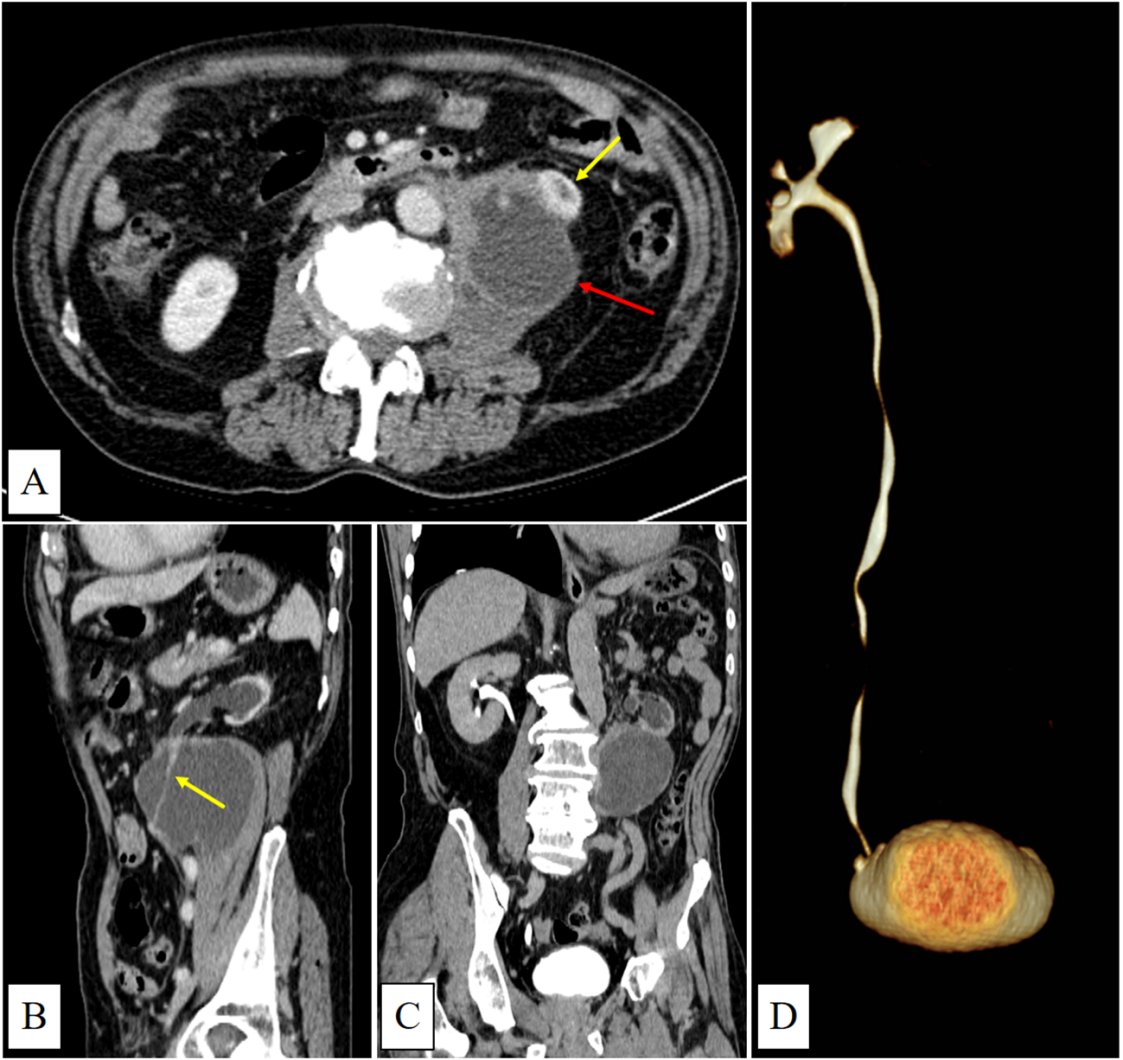
**(A)** (venous phase) shows a predominantly cystic mass with irregular margins and mild enhancement located in the left psoas major region (red arrow). The lower pole of the left kidney is invaded (yellow arrow). **(B)** (sagittal view, venous phase) shows the left ureter running through the mass with mild enhancement(yellow arrow). **(C)** (delayed phase) demonstrates left renal atrophy, dilatation of the left renal pelvis and calyces, and absence of contrast filling within the mass. **(D)** (CTU) shows no contrast enhancement in the left renal pelvis, calyces, or ureter.

CT showed a large cystic-solid mass in the left retroperitoneal and psoas muscle region, with indistinct margins adjoining the left kidney. The left ureter was observed traversing the lesion. Additionally, the left kidney was atrophic with absent excretory function (**Figures 1A–D**).

### Surgery and pathology

The patient underwent laparoscopic resection of the retroperitoneal lesion combined with left nephrectomy on December 9, 2024. Intraoperatively, a large cystic mass was identified within the retroperitoneal space. Ultrasound-guided incision and aspiration of the cyst fluid were performed, revealing dense adhesions between the ureter and cyst wall. Histopathological analysis confirmed invasive high-grade urothelial carcinoma with a Ki-67 proliferation index exceeding 90%, along with tumor infiltration of the retroperitoneal cyst wall. Surgical margins were positive.

### Follow up

The patient’s blood pressure was 106/78 mmHg on postoperative day one, and he did not receive adjuvant radiotherapy or chemotherapy after discharge. Six months later, the patient developed tumor progression at the surgical site, complicated by an intestinal fistula and retroperitoneal infection. On May 31, 2025, he underwent left hemicolectomy, partial small bowel resection, and transverse colostomy. Histopathological examination confirmed metastatic urothelial carcinoma involving the descending colon and small intestine. Tragically, a follow-up abdominal CT scan performed 20 days after surgery revealed widespread metastases involving the liver, peritoneum, omentum, abdominal wall, and lumbar vertebrae.

## Discussion

The clinical manifestations of urinomas vary from subtle signs to acute abdominal presentations, often leading to delayed diagnosis. When urine is reabsorbed through the peritoneum, patients may develop symptoms mimicking acute renal failure (4). Diagnostic imaging modalities for urinomas include ultrasound, intravenous urography, contrast-enhanced abdominal CT, and PET-CT. Delayed-phase CT imaging (obtained 5–20 minutes after contrast administration) that demonstrates iodinated urine surrounding the urinary tract is crucial for diagnosis (5). Intraoperative retrograde pyelography can further assist in localizing the site of rupture (6).

According to literature reviews, simple urinomas typically appear as iodinated urine collections surrounding the urinary tract on delayed-phase CT scans. When progressing to abscess formation, they may exhibit peripherally enhancing rims, but usually lack irregularly thickened solid components (2, 5). In our case, CT imaging revealed a large cystic-solid mass occupying most of the left psoas major muscle, with the left ureter traversing it intact—an atypical presentation possibly related to chronic urine leakage from a microscopic ureteral rupture. Additionally, no iodinated urine was observed within the cystic lesion on the delayed-phase CT, which is consistent with impaired excretory function of the left kidney. The patient’s preoperative hypertension may have been secondary to activation of the renin-angiotensin-aldosterone system (RAAS) in response to renal ischemia (7).

The initial misdiagnosis of a psoas muscle tumor in this case can be attributed to several factors. First, the patient was asymptomatic, and both urinalysis and urine cytology yielded negative results. Second, the understanding of the classic imaging features, such as the “beak sign” (8) and “phantom organ sign” (9) was relatively narrow, along with insufficient recognition of urinoma imaging features, which contributed to the diagnostic error. In this case, multiplanar CT images showed sharp angles between the lesion margins and the lateral border of the left psoas major, while the continuity of the left ureter was preserved, leading to mislocalization. Additionally, extensive tumor infiltration by urothelial carcinoma masked the relatively small primary lesion within the left ureter, resulting in its oversight. Finally, loss of excretory function in the left kidney delayed iodinated urine extravasation, further complicating the interpretation of contrast-enhanced CT findings.

The patient experienced rapid disease progression within six months post-surgery, despite initial symptomatic improvement. Due to the tumor’s high invasiveness and severe adhesion to surrounding tissues, complete R0 resection could not be achieved during the initial operation. Additionally, the use of an ultrasonic scalpel to incise the lesion and aspirate cyst fluid intraoperatively may have contributed to tumor dissemination and accelerated disease progression. This case highlights the need for clinicians to maintain heightened vigilance when evaluating potential cases of urinoma. Patients may be entirely asymptomatic, and the presence of fluid-density collections around the kidney or ureter on imaging should prompt consideration of urinoma. If irregular enhancing solid components are present, concurrent tumor infiltration should be suspected. Notably, in cases of impaired renal function, delayed-phase CT may fail to reveal iodinated urine extravasation, reducing diagnostic sensitivity. Preoperative evaluation should include a thorough assessment of the feasibility of achieving complete resection. When surgery is indicated, preservation of tumor capsule integrity is essential to minimize the risk of peritoneal or retroperitoneal spread. Furthermore, different treatment strategies significantly impact patient survival outcomes, more effective therapeutic strategies are urgently needed to enhance patients’ quality of life and improve prognosis (10).

## Conclusion

This case of retroperitoneal urinoma secondary to metastatic urothelial carcinoma underscores the complexity of diagnosing and treating urothelial malignancies. It highlights the importance of comprehensive imaging evaluation and the challenges associated with poor prognostic outcomes.

## Data Availability

The original contributions presented in the study are included in the article/supplementary material. Further inquiries can be directed to the corresponding author.
